# Supplementary material for: A third vaccination with a single T cell epitope confers protection in a murine model of SARS-CoV-2 infection
Source: Nat Commun. 2022 Jul 8;13:3966. doi: 10.1038/s41467-022-31721-6 (PMC9267705; doi:10.1038/s41467-022-31721-6)
Supplement: Supplementary file 1 — Supplementary Information [file 41467_2022_31721_MOESM1_ESM.pdf]

Supplementary Information

A third vaccination with a single T cell epitope confers protection in a murine model of SARS-CoV-2 infection

Iris N. Pardieck[1], Tetje C. van der Sluis[1*], Esmé T.I. van der Gracht[1*], Dominique M.B. Veerkamp[1], Felix M. Behr[1], Suzanne van Duikeren[1], Guillaume Beyrend[1], Jasper Rip[1], Reza Nadafi[1], Elham Beyranvand Nejad[1], Nils Mülling[1], Dena J. Brasem[1], Marcel G.M. Camps[1], Sebenzile K. Myeni[2], Peter J. Bredenbeek[2], Marjolein Kikkert[2], Yeonsu Kim[3], Luka Cicin-Sain[3], Tamim Abdelaal[4,5], Klaas P.J.M. van Gisbergen[6], Kees L.M.C. Franken[1], Jan Wouter Drijfhout[1], Cornelis J.M. Melief[7], Gerben C.M. Zondag[8], Ferry Ossendorp[1], Ramon Arens[1]

Contents:

Supplementary Figure 1,2,3,4, 5

Supplementary Table 1,2, 3

Supplementary Methods

Supplementary References

Supplementary Figure 1

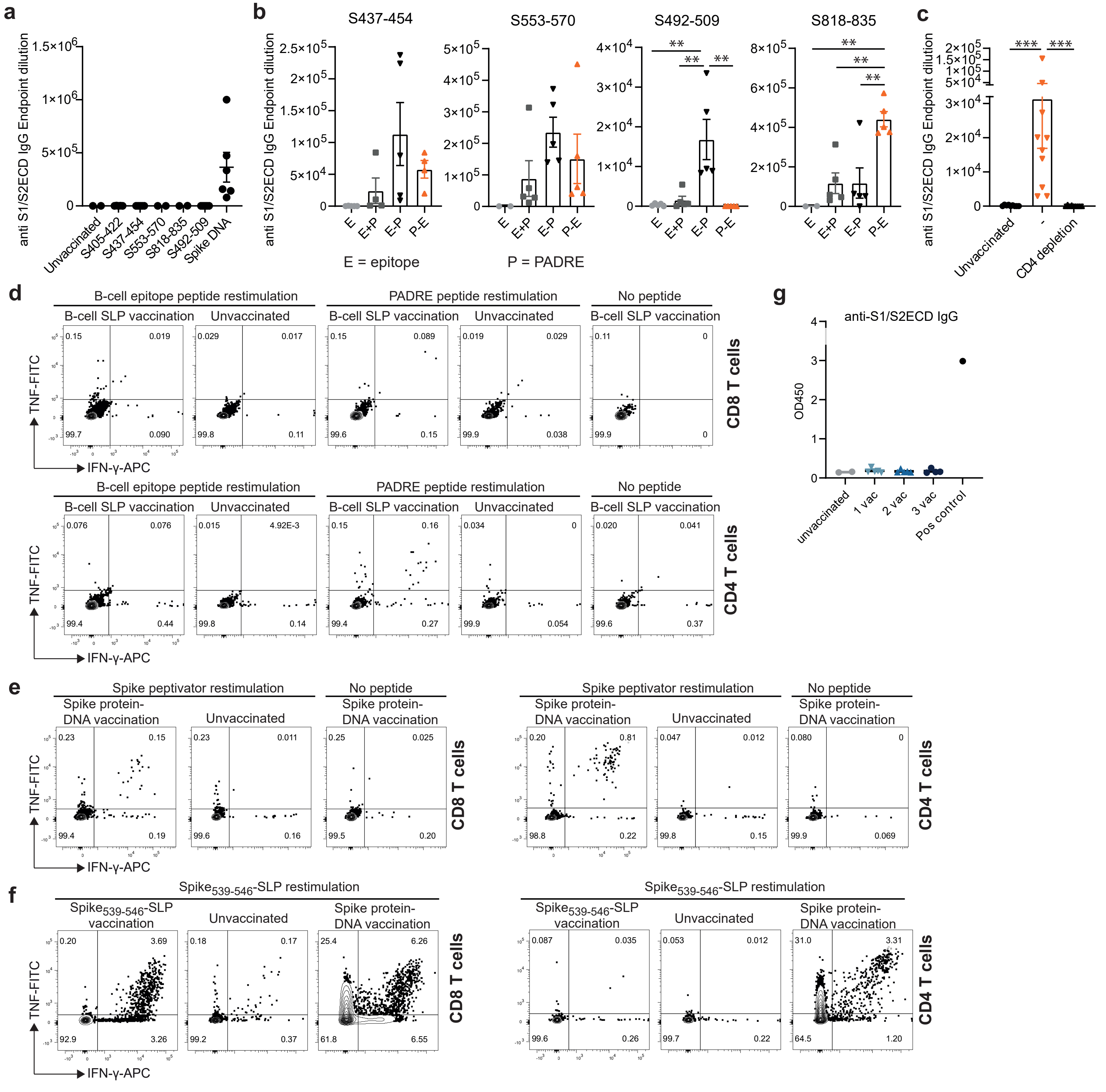

Supplementary Figure 1. Synthetic long peptide (SLP)-based vaccines containing a single B cell epitope require adjuvants to elicit antibodies. (a) C57BL/6 mice were subcutaneously vaccinated on day 0, day 14 and day 28 with SLPs containing linear B cell epitopes adjuvanted with CpG and Inactivated Freund's Adjuvant (IFA). Spike-specific IgG in blood was determined at day 42 after vaccination. Serum from a Spike protein-encoding DNA vaccinated animal was used as a positive control. Results are depicted as the endpoint dilution. Data represented as mean ± SEM. S553-570, S818-835: n=2; S405-422, S437-454, S492-509: n=5; Spike DNA: n=6. **(b)** C57BL/6 mice were vaccinated as described in (a) with SLPs consisting of the B-cell epitope alone (E), the B cell epitope combined with the PADRE peptide (E+P) or C-terminal and N-Terminal PADRE-coupled linear B cell epitopes (E-P, P-E). Spike-specific IgG in blood at day 42 after vaccination. Results are depicted as the endpoint dilution. Data represented as mean ± SEM. Symbols represent individual mice. S437-454: E, E-P: n=5; E+P, P-E: n=4. S553-570: E, n=2, other n=5. S492-509: n=5. S818-835: E, n=2, other n=5. **P=0.006-0.0059. One-way ANOVA with Tukey's multiple comparisons test was performed to determine statistical significance. **(c)** Spike-specific IgG antibodies in blood at day 27 in unvaccinated mice and in $CD4^+$ T cell depleted and proficient mice that were vaccinated with SLPs consisting of PADRE-coupled linear B cell epitopes adjuvanted with CpG and IFA. Results are depicted as the endpoint dilution. Data is represented as mean ± SEM (n=10). ***P=0.001-0.008. Kruskal-Wallis test with multiple comparisons was performed to determine significance. **(d)** Intracellular cytokine production (IFN-γ and TNF) of $CD8^+$ and $CD4^+$ T cells in the spleen at day 35 after PADRE-coupled linear B cell epitope SLP vaccination adjuvanted with CpG and IFA. Splenocytes were restimulated with the SLPs containing linear B cell epitopes coupled to PADRE, the PADRE peptide alone or no peptide. **(e)** Intracellular cytokine production (IFN-γ and TNF) of splenic $CD8^+$ and $CD4^+$ T cells at day 70 after Spike protein DNA vaccination. Splenocytes were restimulated with the S1 peptivator mix or no peptide. **(f)** Intracellular cytokine production of splenic $CD8^+$ and $CD4^+$ T cells at day 35 after $Spike_{539-546}$ SLP vaccination or Spike protein DNA vaccination and re-stimulated with Spike539-546-SLP. **(g)** OD450 values of Spike-specific IgG in blood at day 42 after either 1, 2 or 3 times vaccination with the Spike539-546-SLP , Serum from a Spike-encoding DNA vaccinated animal was used as a positive control. Data represented as mean ± SEM (n=5). Symbols represent individual mice. Source data are provided as Source Data file.

Supplementary Figure 2

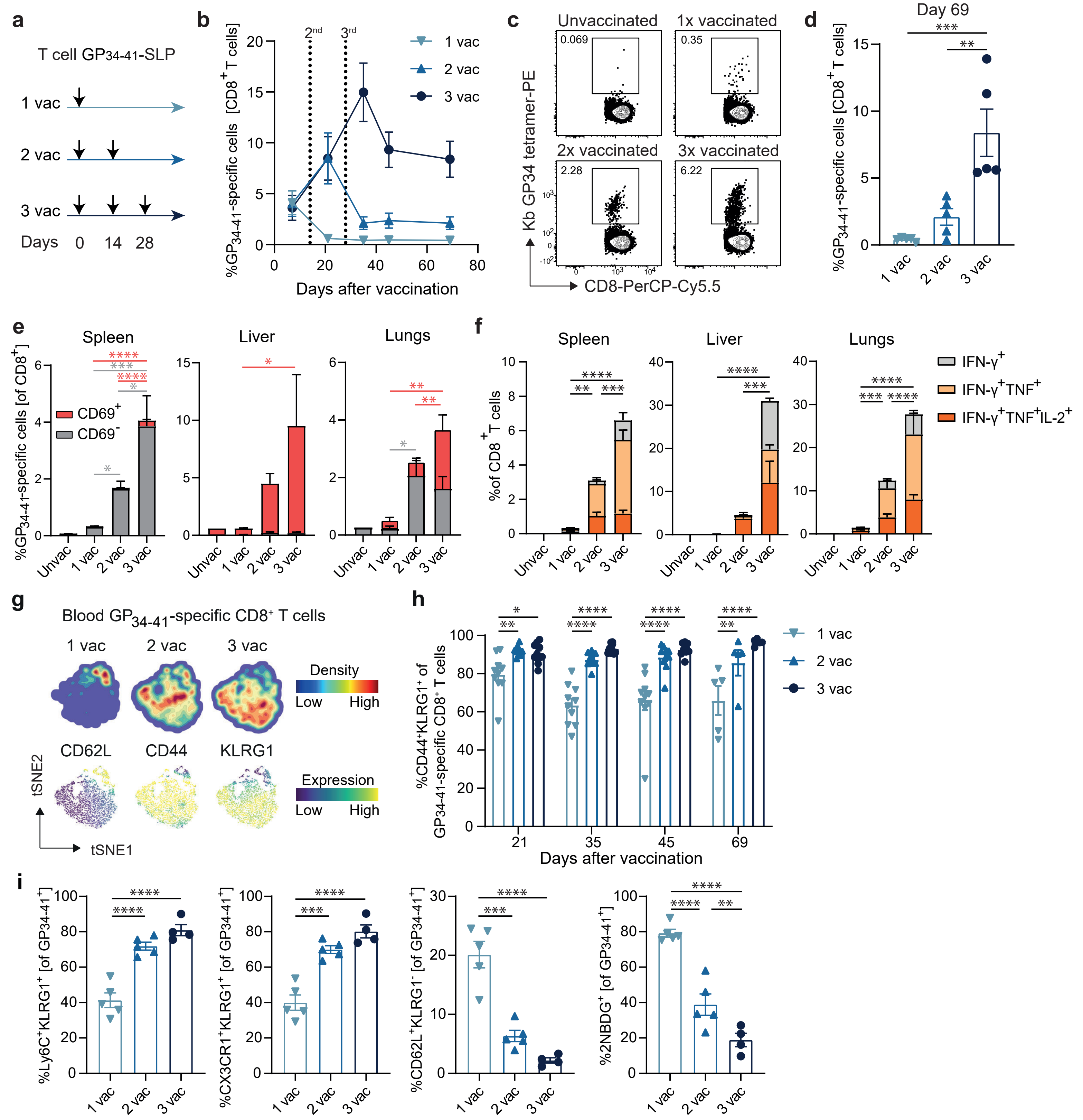

Supplementary Figure 2. A third vaccination with a single T cell epitope augments effector-memory and tissue-resident memory $CD8^+$ T cell formation. (a) C57BL/6 mice were vaccinated with the GP34-41-SLP vaccine adjuvanted with CpG in a prime-boost-boost regimen with 2 week intervals. **(b)** GP34-41-specific $CD8^+$ T cell kinetics in blood at indicated days after vaccination. Data represented as mean ± SEM (n=5 per group). **(c)** Representative flow cytometry plots of GP34-41-specific $CD8^+$ T cells determined by MHC class I tetramer staining. **(d)** GP34-41-specific $CD8^+$ T cells in blood at day 69 after vaccination. Data represented as mean ± SEM (n=5 per group). Symbols represent individual mice. **P=0.0038, ***P=0.0006. **(e)** Frequencies of $CD69^+$ and $CD69^-$ GP34-41-specific $CD8^+$ T cells in the $CD8^+$ T cell population in the spleen, liver and lungs at day 66 after 1, 2 or 3 SLP vaccinations. Data represented as mean + SEM (Spleen 1 vac, 2 vac: n=10, 3 vac: n=9; Liver/Lungs 1 vac, 2 vac: n=5, 3 vac: n=4). *P=0.037-0.0477, **P=0.0049-0.0097, ***P=0.0009, ****P=<0.0001. **(f)** Intracellular cytokine production of $CD8^+$ T cells after restimulation with the GP34-41 peptide epitope on day 66 after SLP vaccination. Data represented as mean + SEM (Spleen 1 vac, 2 vac, n=10, 3 vac, n=9; Liver/Lungs 1 vac, 2 vac: n=5, 3 vac: n=4). **P=0.0043, ***P=0.003-0.005, ****P=<0.0001. **(g)** tSNE maps describing the local probability density of GP34-41-specific $CD8^+$ T cells in the blood stained for CD62L, CD44, KLRG1 at day 71 after 1, 2 and 3 vaccinations. **(h)** Frequencies of $CD44^+KLRG1^+$ GP34-41-specific $CD8^+$ T cells in blood at indicated time points. Data represented as mean ± SEM (Day 21, day 35 and day 45: n=10; Day 69: n=5). Symbols represent individual mice. *P=0.0124-0.0334, **P=0.0011-0.0033, ****P=<0.0001. **(i)** Cell surface marker ($Ly6C^+KLRG1^+$, $CX3CR1^+KLRG1^+$ and $CD62L^+KLRG1^-$) expression and 2-NBDG uptake of splenic GP34-41-specific $CD8^+$ T cells at day 66 post SLP vaccination. Data represented as mean ± SEM (1 vac, 2 vac: n=5; 3 vac: n=4). Symbols represent individual mice. **P=0.024, ***P=0.001-0.002, ****P=<0.0001. One-way ANOVA with Tukey's multiple comparisons test for (**d-f, h, i**). Source data are provided as Source Data file.

Supplementary Figure 3

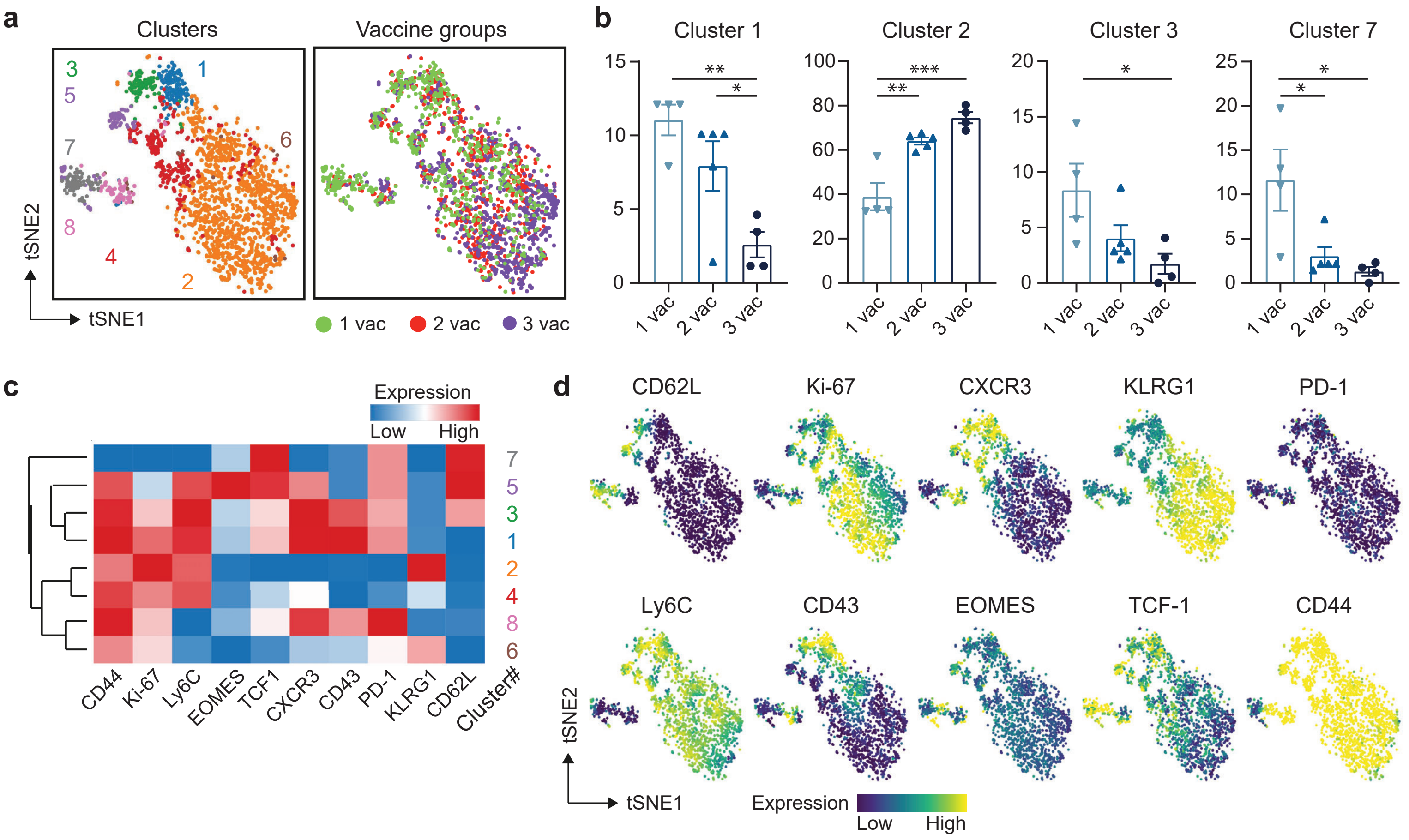

Supplementary Figure 3. Progressive differentiation of vaccine-specific CD8+ T cells after booster vaccination. C57BL/6 mice were vaccinated subcutaneously on day 0 (1st vaccination), day 14 (2nd vaccination) and day 28 (3rd vaccination) with the $GP_{34\text{-}41}$-SLP vaccine adjuvanted with CpG. Following downsampling tSNE analysis, FlowSOM consensus metaclustering with 8 clusters analysis was performed on 695 live CD3$^+$CD8$^+$CD4$^-$CD19$^-$ $Spike_{539\text{-}546}$ tetramer$^+$ cells per group harvested at day 66 after SLP vaccination. Overlay of **(a)** the 8 FlowSOM clusters and vaccination status on the tSNE-map. **(b)** Significant clusters were selected and shown in bar graphs. Data represented as mean ± SEM. Symbols represent individual mice. 1 vac; 2 vac, n=5. 3 vac, n=4. *P=0.0132-0.0428, **P=0.0015-0.0043, ***P=0.0002. One-way ANOVA with Tukey's multiple comparisons test was performed to determine statistical significance. **(c)** Hierarchically clustered heatmap of phenotypes of the clusters shown in (A) – the indicated marker expression is shown per cluster as z-Score of median signal intensity per channel; blue, low expression; red, high expression. **(d)** Expression intensity of cell surface markers (color indication: blue, low expression; yellow, high expression). Source data are provided as a Source Data file.

Supplementary Figure 4

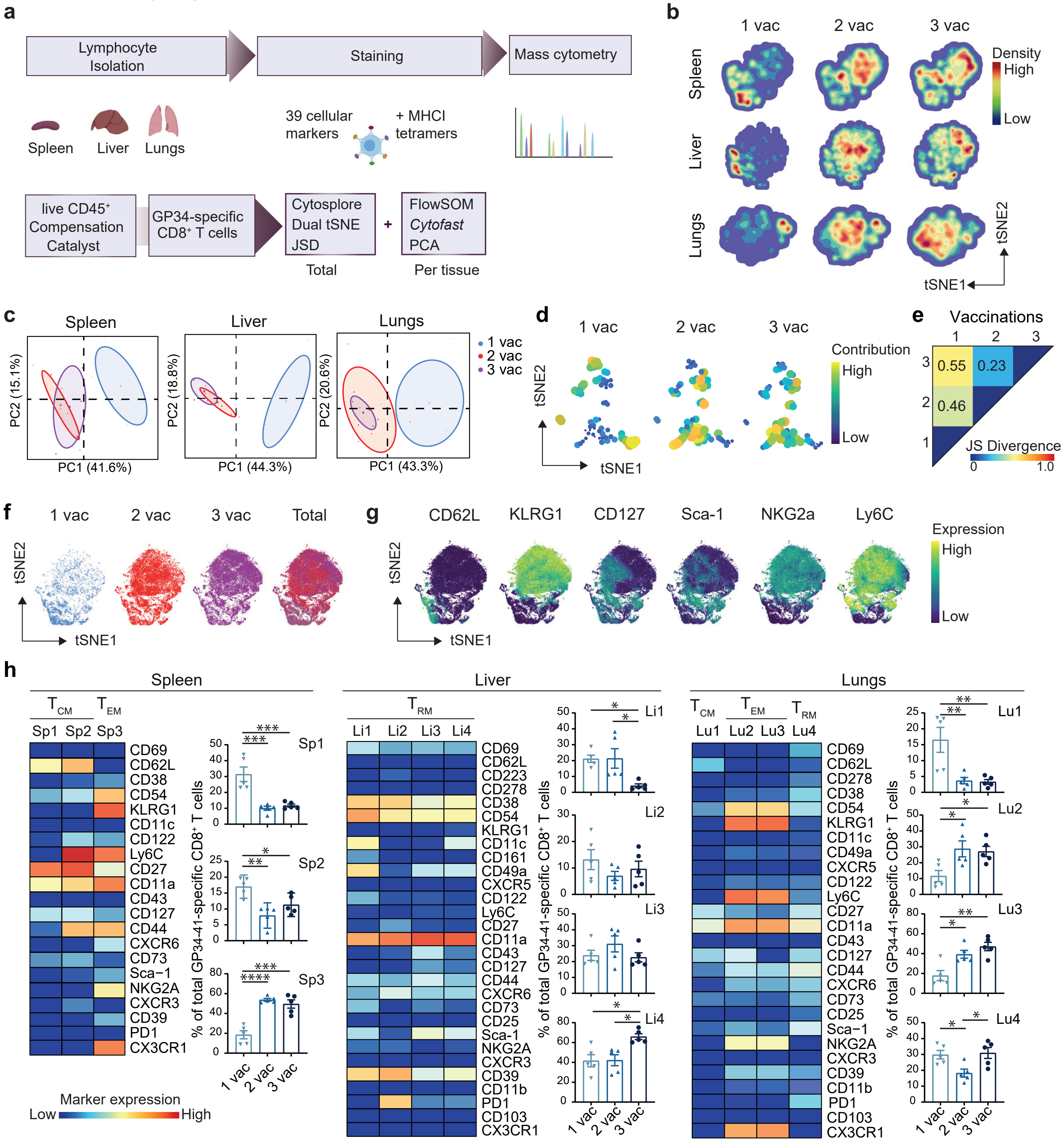

Supplementary Figure 4. Booster vaccination impacts the differentiation of vaccine-specific CD8$^+$ T cells system-wide. (a) Schematic of the mass cytometric analysis of lymphocytes isolated from spleen, liver, and lungs. **(b)** tSNE maps describing the local probability density of GP$_{34\text{-}41}$-specific CD8$^+$ T cells in spleen, liver and lungs stained at day 71 after 1, 2 and 3 vaccinations with the mass cytometry panel. **(c)** Principal Component Analysis illustrating the phenotypic dissimilarity of GP$_{34\text{-}41}$-specific CD8$^+$ T cells per tissue upon multiple vaccinations. **(d)** tSNE maps showing GP$_{34\text{-}41}$-specific CD8$^+$ T cell clusters per vaccination. Clusters with similar composition profiles across samples end up close together in the map. The varying dot size and color in this cluster tSNE map shows the average cluster normal-izedfrequencies per vaccination group. **(e)** Pairwise Jensen-Shannon Divergence plots of the tSNE map obtained from all samples of GP$_{34\text{-}41}$-specific CD8$^+$ T cells grouped by vaccinations. **(f)** tSNE embedding of GP$_{34\text{-}41}$-specific CD8$^+$ T cells isolated from vaccinated mice and multiple tissues in one analysis. Cells are color coded per vaccination. **(g)** Expression intensity of the cell-surface markers on the GP$_{34\text{-}41}$-specific CD8$^+$ T cells. The color of the cells indicates ArcSinh5-transformed expression values for a given marker analyzed. **(h)** Heat maps of GP$_{34\text{-}41}$-specific CD8$^+$ T cell clusters in the spleen, liver and lungs of mice that received multiple vaccinations. Clusters were selected based on their significant difference and categorized in T$_{CM}$, T$_{EM}$ and T$_{RM}$ subsets. Bar graphs indicate the average percentage (± SEM, n=5) of each cluster within the GP$_{34\text{-}41}$-specific CD8$^+$ T-cell population elicited by 1, 2 and 3 vaccinations. Symbols represent individu-al mice. Spleen: *P=0.0302, **P=0.0014, ***P=0.0001-0.0009, ****P=<0.0001; Liver: *P=0.0133-0.0217; Lungs: *P=0.0239-0.0440, **P=0.0015-0.0065. One-way ANOVA with Tukey's multiple comparisons test was performed to determine statistical significance. Source data are provided as a Source Data file.

Supplementary Figure 5

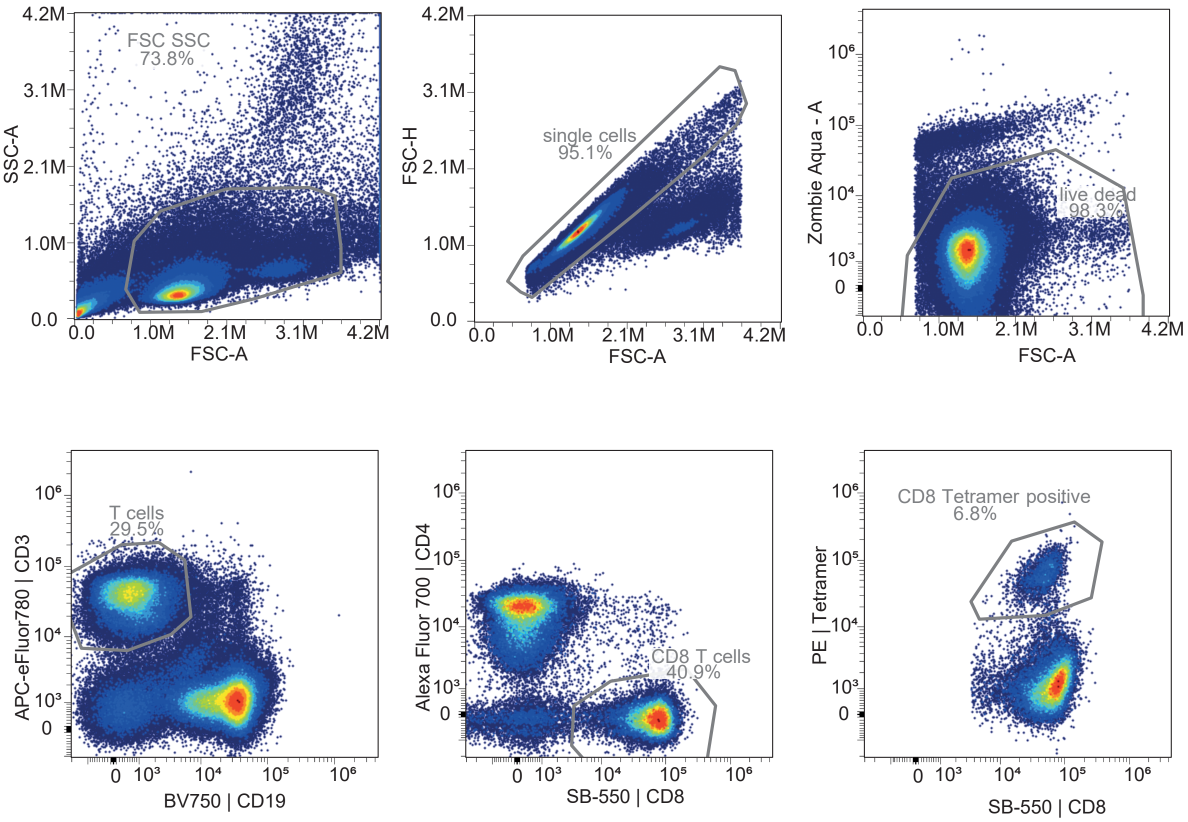

Supplementary Figure 5. Flow cytometry gating strategies. Representative plots show the gating strategy for detecting MHC class I tetramer positive cells depicted. In this sequential gating, cells were first gated on lymphocytes (forward-scatter (FSC-A) vs. side-scatter (SSC-A)) and then on singlets (FSC-A vs. FSC-H). The cells were next analysed for their uptake of Zombie Aqua to exclude dead cells, followed by their expression of CD3 and CD8. Finally, MHC class I tetramer positive cells were gated on the live $CD3^{+}CD8^{+}$ T-cell population.

Table S1. Synthetic long peptides

X = cyclohexylalanine, u = d-Ala, B = amide

PADRE	AKXVAAWTLKAuB
Spike$_{553-570}$	TESNKKFLPFQQFGRDIA
Spike$_{553-570}$-PADRE	TESNKKFLPFQQFGRDIAAKXVAAWTLKAuB
PADRE-Spike$_{553-570}$	AKXVAAWTLKAuTESNKKFLPFQQFGRDIA
Spike$_{818-835}$	PSKPSKRSFIEDLLFNKV
Spike$_{818-835}$-PADRE	PSKPSKRSFIEDLLFNKVAKXVAAWTLKAuB
PADRE-Spike$_{818-835}$	AKXVAAWTLKAuPSKPSKRSFIEDLLFNKV
Spike$_{405-422}$	DEVRQIAPGQTGKIADYN
Spike$_{405-422}$-PADRE	DEVRQIAPGQTGKIADYNAKXVAAWTLKAuB
PADRE-Spike$_{405-422}$	AKXVAAWTLKAuDEVRQIAPGQTGKIADYN
Spike$_{437-454}$	NSNNLDSKVGGNYNYLYR
Spike$_{437-454}$-PADRE	NSNNLDSKVGGNYNYLYRAKXVAAWTLKAuB
PADRE-Spike$_{437-454}$	AKXVAAWTLKAuNSNNLDSKVGGNYNYLYR
Spike$_{492-509}$	LQSYGFQPTNGVGYQPYR
Spike$_{492-509}$-PADRE	LQSYGFQPTNGVGYQPYRAKXVAAWTLKAuB
PADRE-Spike$_{492-509}$	AKXVAAWTLKAuLQSYGFQPTNGVGYQPYR
Spike$_{539-546}$	IKNQCVNFNFNGLTGTGVLTESNK
GP$_{34-41}$	KAVYNFATCGIFALIS
OVA	SMLVLLPDEVSGLEQLESIINFEKLTEWTS

Table S2. Flow Cytometry antibodies and MHC tetramers

Antibody	Fluorophore	Clone	Supplier	Catalogue number	Dilution
CD3	APC-eF780	17A2	Thermofisher	47-0032-82	1:100
CD3	PE	145-2C11	Thermofisher	12-0031-83	1:200
CD4	FITC	RM4-4	Thermofisher	11-0043-85	1:400
KLRG1	PE-Cy7	2F1	Thermofisher	25-5893-82	1:100
KLRG1	BV785	2F1	Biolegend	138429	1:100
IFNγ	APC	XMG1.2	Thermofisher	17-7311-82	1:400
CD3	BV510	145-2C11	BD Biosciences	563024	1:150
CD69	BV711	H1.2F3	BD Biosciences	104537	1:100
PD-1	BUV615	J43	BD Biosciences	752299	1:100
TCF1	AF647	S33-966	BD Biosciences	566693	1:100
CD4	AF700	RM4-5	BioLegend	100536	1:400
CD8a	BV605	53-6.7,	BioLegend	100743	1:1000
CD8a	SparkBlue550	53-6.7	Biolegend	100779	1:400
CD38	PE	90	Thermofisher	12-0381-81	1:200
CD44	BV786	IM7	BioLegend	103059	1:100
CD44	PacB	IM7	Biolegend	103019	1:200
CD44	APC-Cy7	IM7	BD Biosciences	560568	1:600
CD62L	BV421	MEL-14	BioLegend	104435	1:300
CD62L	BUV395	MEL-14	BD Biosciences	740218	1:1600
CD62L	BV510	MEL-14	Biolegend	104441	1:800
CX3CR1	BV785	SA011F11	BioLegend	149029	1:1600
Ly6C	FITC	HK1.4	BioLegend	128006	1:50
Ly6C	PacB	HK1.4	Biolegend	128013	1:600
TNF	FITC	MP6-XT22	BioLegend	506304	1:1000

Ki-67	BV605	16A8	BioLegend	652413	1:800
CXCR3	BV650	CXCR3-173	BioLegend	126531	1:150
CD19	BV750	6D5	BioLegend	115561	1:300
CD43	PerCP-Cy5.5	1B11	BioLegend	121223	1:200
Zombie Aqua	BV510	-	BioLegend	423101	1:800
Eomes	PE-Cy7	Dan11mag	Invitrogen	25-4875-82	1:100
CD3	FITC	145-2C11	Thermofisher	11-0031-85	1:100
IL-2	PE	JES6-5H4	Thermofisher	12-7021-82	1:200
**MHC Tetramer**	**Fluorophore**	**Peptide**	**Supplier**		**Dilution**
MHC class I H2-Kb tetramer SARS-CoV-2 Spike$_{539-546}$	APC	VNFNFNGL	LUMC Tetramer Facility		1:100
MHC class I H2-Kb tetramer LCMV GP$_{34-41}$	PE	AVYNFATC	LUMC Tetramer Facility		1:200
MHC class I H2-Kb tetramer Ovalbumin OVA$_{257-264}$	APC	SIINFEKL	LUMC Tetramer Facility		1:400
MHC class II I-A(b) tetramer PADRE	PE	AKFVAAWTLKAA	NIH Tetramer Core Facility		1:200

Table S3. CyTOF Mass Cytometry Panel. Anti-mouse monoclonal antibodies used for staining of cells for mass cytometry analysis. Antibodies were either purchased pre-conjugated, or antibodies were conjugated to the indicated lanthanide metal isotopes.

Antibody	Clone	Metal	Pre-conjugated	Company	Cat no	Cat no metal (Fluidigm)
**Anti-PE**	PE001	165 Ho	x	Fluidigm	3165015B	
**Anti-APC**	APC003	176 Yb	x	Fluidigm	3176007B	
**CD3e**	145-2C11	172 Yb		eBioscience	14-0031-86	201172A
**CD4**	RM4-5	145 Nd	x	Fluidigm	3145002B	
**CD8a**	53-6.7	168 Er	x	Fluidigm	3168003B	
**CD8b**	YTS156.7.7	194 Pt		BioLegend	126602	201194
**CD11a**	M17/4	160 Gd		eBioscience	16-0111-82	201160A
**CD11b**	M1/70	154 Sm	x	Fluidigm	3154006B	
**CD11c**	N418	167 Er		eBioscience	14-0114-85	201167A
**CD19**	6D5	Qdot655: 112/114 Cd	x	ThermoFisher	Q10379	
**CD25**	3C7	150 Nd	x	Fluidigm	3150002B	
**CD27**	LG.3A10	158 Gd		eBioscience	14-0272-82	201158A
**CD38**	90	163 Dy		eBioscience	14-0381-85	201163A
**CD39**	24DMS1	152 Sm		eBioscience	14-0391-82	201152A
**CD43**	1B11	115 In		BioLegend	121202	
**CD44**	IM7	142 Nd		eBioscience	14-0441-86	201142A
**CD45**	30-F11	89Y	x	Fluidigm	3089005B	
**CD49a**	Ha31/8	151 Eu		BD Biosciences	555001	201151A
**CD54**	YN1/1.7.4	164 Dy		BioLegend	116102	201164A
**CD62L**	MEL-14	169 Tm		BioLegend	104443	201169A
**CD69**	H1.2F3	143 Nd	x	Fluidigm	3143004B	
**CD73**	TY/23	148 Nd		BD Biosciences	550738	201148A
**CD86**	GL1	171 Yb		eBioscience	14-0862-85	201171A
**CD103**	2.E7	173 Yb		eBioscience	14-1031-85	201173A
**CD122**	TM-b1	155 Gd		eBioscience	14-1222-85	201155A
**CD127**	A7R34	175 Lu	x	Fluidigm	3175006B	
**CD160**	7H1	209 Bi		BioLegend	143002	
**CD161**	PK136	170 Er	x	Fluidigm	3170002B	
**CD223**	eBioC9B7W	161 Dy		eBioscience	14-2231-85	201161A
**CD278**	7E.17G9	162 Dy		eBioscience	14-9942-85	201162A
**CX3CR1**	SA011F11	174 Yb		BioLegend	149002	201174A
**CXCR3**	CXCR3-173	149 Sm		eBioscience	16-1831-85	201149A
**CXCR5**	L138D7	153 Eu		BioLegend	145502	201153A
**CXCR6**	SA051D1	144 Nd		BioLegend	151102	201144A
**FR4**	TH6	198 Pt		BioLegend	125102	201198
**KLRG1**	2F1	166 Er		eBioscience	16-5893-85	201166A
**Ly6C**	HK1.4	156 Gd		eBioscience	16-5932-85	201156A
**NKG2A**	20d5	147 Sm		eBioscience	16-5896-85	201147A
**PD-1**	29F.1A12	159 Tb	x	Fluidigm	3159024B	
**Sca-1**	D7	141 Pr		BioLegend	108135	201141A
**TCRgd**	eBioGL3	146 Nd		eBioscience	14-5711-85	201146A

Supplementary Methods

Mass cytometry and analysis.

Metal-conjugated antibodies were either purchased from Fluidigm or were generated by conjugation of lanthanide metal isotopes to anti-mouse antibodies using the Maxpar X8 Polymer method according to the manufacturer's protocol (Fluidigm). Cisplatins 194 and 198 and Bismuth 209 were conjugated to anti-mouse monoclonal antibodies using protocols previously described (1, 2). All in-house conjugated antibodies were diluted to 0.5 mg/ml in antibody stabilizer supplemented with 0.05% sodium azide (Candor Biosciences). Serial dilution staining was performed on mouse lymphocytes to determine appropriate antibody dilution.

The CyTOF staining was performed as described elsewhere (3). In brief, around 3×10^6 cells per sample were stained for CyTOF analysis. First, cells were stained in FACS buffer with PE and APC labelled tetramers and incubated for 30 minutes on ice. Cells were washed with Maxpar Cell Staining buffer (201068, Fluidigm) and subsequently incubated for 20 minutes with 1 µM Interchalator-Rh (201103A, Fluidigm) in staining buffer. Next, aspecific binding was prevented by incubating cells with Fc blocking solution and mouse serum for 15 minutes. Anti-PE and anti-APC were added and incubated for 45 minutes. The antibody mix was added and incubated for an additional 45 minutes. After washing the cells, samples were incubated overnight with 25nM Intercalator-Ir (201192A, Fluidigm) in Maxpar Fix and Perm Buffer (201067, Fluidigm). Cells were pelleted in staining buffer and measured within one week. Before measuring, EQ™ Four Element Calibration Beads (201078, Fluidigm) were added in a 1:10 ratio. Mass cytometry data analysis focused on the antigen-specific $CD8^+$ T cells. For the selection, we set our gating strategy to live single cells, positive for CD45, and excluded reference beads. The live $CD45^+$ gated files were compensated using Catalyst (4). Subsequently, MHC class I tetramer-specific $CD8^+$ T cells were selected in FlowJo for subsequent analysis. Next, marker expression was ArcSinh5 transformed and subjected to dimensionality reduction analyses and cluster identification using Cytosplore (5) or FlowSOM (6). For Cytosplore analysis, samples were analysed by hierarchical stochastic neighbor embedding (HSNE) (7) based on approximated t-distributed stochastic neighbor embedding (A-tSNE) (8). The similarity between tSNE maps was quantified using the Jensen-Shannon (JS) divergence as previously described (3). The JS divergence values ranged from 0 (indicating identical distributions) to 1 (indicating disjoint distributions). The dual tSNE analysis was performed to quantify the individual samples similarity based on the clusters composition as previously described (3, 9). FlowSOM was used for the identification of vaccination and tissue-specific clusters. Using FlowSOM, 14 clusters were identified per analysis. Subsequently, Cytofast (10, 11) was used for visualization and quantification of cell clusters. T_{RM} ($CD62L^-$ $CD69^+$), T_{CM} ($CD62L^+$ $CD69^-$) and T_{EM} ($CD62L^-CD69^-$) $CD8^+$ T cell clusters were selected by expression of CD62L and CD69. Visualization of heatmaps and selection of clusters based on the size of the cluster (abundance of at least >5% of total) and significance was performed as previously described (3).

Supplementary References

1. Mei HE, Leipold MD, Maecker HT. Platinum-conjugated antibodies for application in mass cytometry. Cytometry A. 2016;89(3):292-300.
2. Spitzer MH, Carmi Y, Reticker-Flynn NE, Kwek SS, Madhireddy D, Martins MM, et al. Systemic Immunity Is Required for Effective Cancer Immunotherapy. Cell. 2017;168(3):487-502.e15.
3. van der Gracht ETI, Beyrend G, Abdelaal T, Pardieck IN, Wesselink TH, van Haften FJ, et al. Memory CD8+ T cell heterogeneity is primarily driven by pathogen-specific cues and additionally shaped by the tissue environment. iScience. 2021;24(1):101954.
4. Chevrier S, Crowell HL, Zanotelli VRT, Engler S, Robinson MD, Bodenmiller B. Compensation of Signal Spillover in Suspension and Imaging Mass Cytometry. Cell Systems. 2018;6(5):612-20.e5.
5. Höllt T, Pezzotti N, van Unen V, Koning F, Eisemann E, Lelieveldt B, et al. Cytosplore: Interactive Immune Cell Phenotyping for Large Single-Cell Datasets. Computer Graphics Forum. 2016;35(3):171-80.
6. Van Gassen S, Callebaut B, Van Helden MJ, Lambrecht BN, Demeester P, Dhaene T, et al. FlowSOM: Using self-organizing maps for visualization and interpretation of cytometry data. Cytometry A. 2015;87(7):636-45.
7. van Unen V, Höllt T, Pezzotti N, Li N, Reinders MJT, Eisemann E, et al. Visual analysis of mass cytometry data by hierarchical stochastic neighbour embedding reveals rare cell types. Nature Communications. 2017;8(1):1740.
8. Pezzotti N, Lelieveldt BPF, Van Der Maaten L, Hollt T, Eisemann E, Vilanova A. Approximated and User Steerable tSNE for Progressive Visual Analytics. IEEE Trans Vis Comput Graph. 2017;23(7):1739-52.
9. van Unen V, Li N, Molendijk I, Temurhan M, Hollt T, van der Meulen-de Jong AE, et al. Mass Cytometry of the Human Mucosal Immune System Identifies Tissue- and Disease-Associated Immune Subsets. Immunity. 2016;44(5):1227-39.
10. Beyrend G, Stam K, Hollt T, Ossendorp F, Arens R. Cytofast: A workflow for visual and quantitative analysis of flow and mass cytometry data to discover immune signatures and correlations. Comput Struct Biotechnol J. 2018;16:435-42.
11. Beyrend G, Stam K, Ossendorp F, Arens R. Visualization and Quantification of High-Dimensional Cytometry Data using Cytofast and the Upstream Clustering Methods FlowSOM and Cytosplore. J Vis Exp. 2019(154).
