# Supplementary material for: A third vaccination with a single T cell epitope confers protection in a murine model of SARS-CoV-2 infection
Source: Nat Commun. 2022 Jul 8;13:3966. doi: 10.1038/s41467-022-31721-6 (PMC9267705; doi:10.1038/s41467-022-31721-6)
Supplement: Supplementary file 3 — Reporting Summary [file 41467_2022_31721_MOESM3_ESM.pdf]

nature portfolio

Corresponding author(s): Ramon Arens

Last updated by author(s): May 10, 2022

Reporting Summary

Nature Portfolio wishes to improve the reproducibility of the work that we publish. This form provides structure for consistency and transparency in reporting. For further information on Nature Portfolio policies, see our Editorial Policies and the Editorial Policy Checklist.

Statistics

For all statistical analyses, confirm that the following items are present in the figure legend, table legend, main text, or Methods section.

n/a	Confirmed	
☐	☒	The exact sample size (*n*) for each experimental group/condition, given as a discrete number and unit of measurement
☐	☒	A statement on whether measurements were taken from distinct samples or whether the same sample was measured repeatedly
☐	☒	The statistical test(s) used AND whether they are one- or two-sided<br>*Only common tests should be described solely by name; describe more complex techniques in the Methods section.*
☒	☐	A description of all covariates tested
☐	☒	A description of any assumptions or corrections, such as tests of normality and adjustment for multiple comparisons
☐	☒	A full description of the statistical parameters including central tendency (e.g. means) or other basic estimates (e.g. regression coefficient) AND variation (e.g. standard deviation) or associated estimates of uncertainty (e.g. confidence intervals)
☐	☒	For null hypothesis testing, the test statistic (e.g. *F*, *t*, *r*) with confidence intervals, effect sizes, degrees of freedom and *P* value noted<br>*Give P values as exact values whenever suitable.*
☒	☐	For Bayesian analysis, information on the choice of priors and Markov chain Monte Carlo settings
☐	☐	For hierarchical and complex designs, identification of the appropriate level for tests and full reporting of outcomes
☒	☐	Estimates of effect sizes (e.g. Cohen's *d*, Pearson's *r*), indicating how they were calculated

Our web collection on statistics for biologists contains articles on many of the points above.

Software and code

Policy information about availability of computer code

Data collection	BD FACSDiva Software (version 9, BD Biosciences), SpectroFlo (version 3, Cytek)
Data analysis	FlowJo v10 (Treestar), Prism 8 (Graphpad), OMIQ data analysis, Cytofast analysis (v1.6.0)

For manuscripts utilizing custom algorithms or software that are central to the research but not yet described in published literature, software must be made available to editors and reviewers. We strongly encourage code deposition in a community repository (e.g. GitHub). See the Nature Portfolio guidelines for submitting code & software for further information.

Data

Policy information about availability of data

All manuscripts must include a data availability statement. This statement should provide the following information, where applicable:
- Accession codes, unique identifiers, or web links for publicly available datasets
- A description of any restrictions on data availability
- For clinical datasets or third party data, please ensure that the statement adheres to our policy

All data associated with this study are present in the paper or the Supplementary Information. Source data are provided with this paper. The different SARS-CoV-2 variants used in this paper can be found in the GISAID database ID: EPI_ISL_451934 and the GenBank database: accession number MT705206.1.

Field-specific reporting

Please select the one below that is the best fit for your research. If you are not sure, read the appropriate sections before making your selection.

☒ Life sciences ☐ Behavioural & social sciences ☐ Ecological, evolutionary & environmental sciences

For a reference copy of the document with all sections, see nature.com/documents/nr-reporting-summary-flat.pdf

Life sciences study design

All studies must disclose on these points even when the disclosure is negative.

Sample size	Sample size were chosen based on prior research conducted in our laboratory to provide sufficient numbers of mice in each group to obtain informative results and perform statistical testing. For all performed experiments, 3-12 mice per group were sufficient to detect differences between groups with a power value of 0.8 and a 5% significance threshold.
Data exclusions	No data points were excluded from data sets.
Replication	The immunogenicity studies described in Figure 1, 2, 3, 4, Supplementary Figure 1, 2, 3 were performed twice. The SARS-CoV-2 challenge experiments were performed once. The experiments in Figure 5 and Supplementary Figure 4 were performed once.
Randomization	The allocation into experimental groups was random in all (animal) experiments.
Blinding	Blinding was not performed in most of the experiments of this study, as experimental observations (e.g. cell surface phenotypes, enumerations), would be consistent irrespective of blinding. During the SARS-CoV-2 challenge experiments, the experimental groups were blinded for the researcher that observed and weighed the mice. During the other immunogenicity studies, where the outcomes where cell surface phenotypes, cell numbers and antibody titrations, no blinding was used. The virus neutralization studies were completely blinded.

Reporting for specific materials, systems and methods

We require information from authors about some types of materials, experimental systems and methods used in many studies. Here, indicate whether each material, system or method listed is relevant to your study. If you are not sure if a list item applies to your research, read the appropriate section before selecting a response.

Materials & experimental systems

n/a	Involved in the study
☐	☒ Antibodies
☐	☒ Eukaryotic cell lines
☒	☐ Palaeontology and archaeology
☐	☒ Animals and other organisms
☒	☐ Human research participants
☒	☐ Clinical data
☒	☐ Dual use research of concern

Methods

n/a	Involved in the study
☒	☐ ChIP-seq
☐	☒ Flow cytometry
☒	☐ MRI-based neuroimaging

Antibodies

Antibodies used

Flow cytometry:
Thermofisher: CD3-FITC (clone 145-2C11, cat 11-0031-85, 1:100), CD3-APC-eF780 (clone 17A2, cat 47-0032-82, 1:100), CD3-PE (clone 145-2C11, cat 12-0031-83, 1:200), CD4-FITC (clone RM4-4, cat 11-0043-85, 1:400), KLRG1-PeCy7 (clone 2F1, cat 25-5893-82, 1:100), IFN-y-APC (clone XMF1.2, cat 17-7311-82, 1:400), IL-2-PE (clone JES6-5H4, cat 12-7021-82, 1:200), CD38-PE (clone 90, cat 12-0381-82, 1:200)
BD Biosciences: CD3-BV510 (clone 145-2C11, cat 563024, 1:150), CD69-BV711 (clone H1.2F3, cat 104537, 1:100), PD-1-BUV615 (clone J43, cat 752299, 1:100), TCF1-AF647 (clone S33-966, cat 566693, 1:100), CD44-APC-Cy7 (clone IM7, cat 560568, 1:600), CD62L-BUV395 (clone MEL-14, cat 740218, 1:1600)
BioLegend: KLRG1-BV785 (clone 2F1, cat 138429, 1:100), CD4-AF700 (clone RM4-5, cat 100536, 1:400), CD8a-BV605 (clone 53-6.7, cat 100743, 1:1000), CD8a-SparkBlue550 (clone 53-6.7, cat 100779, 1:400), CD44-BV786 (clone IM7, cat 103059, 1:100), CD44-PacB (clone IM7, cat 103019, 1:200), CD62L-BV421 (clone MEL-14, cat 104435, 1:300), CD62L-BV510 (clone MEL-14, cat 104441, 1:800), CX3CR1-BV785 (clone SA011F11, cat 149029, 1:1600), Ly6C-FITC (clone HK1.4, cat 128006, 1:50), Ly6C-PacB (clone HK1.4, cat 128013, 1:600), TNF-FITC (clone MP6-XT22, cat 506304, 1:1000), Ki-67-BV605 (clone 16A8, cat 652413, 1:800), CXCR3-BV650 (clone CXCR3-173, cat 126531, 1:150), CD19-BV750 (clone 6D5, cat 115561, 1:300), CD43-PerCP-Cy5.5 (clone 1B11, cat 121223, 1:200), Zombie Aqua (cat 423101, 1:800).
Invitrogen: EOMES-PE-Cy7 (clone Dan11mag, cat 25-4875-82, 1:100)
LUMC Tetramer Facility: MHC class I tetramers: Spike539-546 tetramer-APC (1:100), GP34-41 tetramer-PE (1:200), OVA257-264 tetramer-APC (1:400)
NIH tetramer core facility: MHC class II tetramer for the epitope (AKFVAAWTLKAA)-PE (1:200)

	ELISA:1:4000 dilution of horse radish peroxidase (HRP) conjugated anti-mouse IgG secondary antibody (SouthernBiotech, cat. 1030-05)

Validation

Most antibodies used are commercially available and have been validated by the respective suppliers. Their validation data are available on the manufacturers websites, as listed below:
CD3:
https://www.thermofisher.com/antibody/product/CD3-Antibody-clone-17A2-Monoclonal/47-0032-82
https://www.thermofisher.com/antibody/product/CD3e-Antibody-clone-145-2C11-Monoclonal/12-0031-82
https://www.bdbiosciences.com/en-ca/products/reagents/flow-cytometry-reagents/research-reagents/single-color-antibodies-ruo/bv510-hamster-anti-mouse-cd3e.563024
https://www.thermofisher.com/antibody/product/CD3e-Antibody-clone-145-2C11-Monoclonal/11-0031-82

CD4:
https://www.thermofisher.com/order/genome-database/dataSheetPdf?producttype=antibody&productsubtype=antibody_primary&productId=11-0043-85&version=65
https://www.biolegend.com/fr-ch/products/alexa-fluor-700-anti-mouse-cd4-antibody-3386

CD8a:
https://www.biolegend.com/it-it/products/brilliant-violet-605-anti-mouse-cd8a-antibody-7636
https://www.biolegend.com/en-us/search-results/spark-blue-550-anti-mouse-cd8a-antibody-18489

CD19:
https://www.biolegend.com/de-at/products/brilliant-violet-750-anti-mouse-cd19-antibody-18030

KLRG1:
https://www.thermofisher.com/antibody/product/KLRG1-Antibody-clone-2F1-Monoclonal/25-5893-82
https://www.biolegend.com/nl-be/products/brilliant-violet-785-anti-mouse-human-klrg1-mafa-antibody-13682
IFN-γ:
https://www.thermofisher.com/antibody/product/IFN-gamma-Antibody-clone-XMG1-2-Monoclonal/17-7311-82

CD38:
https://www.thermofisher.com/antibody/product/CD38-Antibody-clone-90-Monoclonal/12-0381-82

CD69:
https://www.biolegend.com/en-gb/products/brilliant-violet-711-anti-mouse-cd69-antibody-12139

PD-1:
https://www.bdbiosciences.com/en-nz/products/reagents/flow-cytometry-reagents/research-reagents/single-color-antibodies-ruo/buv615-hamster-anti-mouse-cd279-pd-1.752299

TCF1:
https://www.bdbiosciences.com/en-us/products/reagents/flow-cytometry-reagents/research-reagents/single-color-antibodies-ruo/alexa-fluor-647-mouse-anti-tcf-7-tcf-1.566693

CD44:
https://www.biolegend.com/en-us/products/brilliant-violet-785-anti-mouse-human-cd44-antibody-7959
https://www.biolegend.com/de-at/search-results/pacific-blue-anti-mouse-human-cd44-antibody-3099
https://www.bdbiosciences.com/en-nz/products/reagents/flow-cytometry-reagents/research-reagents/single-color-antibodies-ruo/apc-cy-7-rat-anti-mouse-cd44.560568

CD62L:
https://www.biolegend.com/en-us/search-results/brilliant-violet-421-anti-mouse-cd62l-antibody-7164
https://www.bdbiosciences.com/en-ca/products/reagents/flow-cytometry-reagents/research-reagents/single-color-antibodies-ruo/buv395-rat-anti-mouse-cd62l.740218
https://www.biolegend.com/de-at/products/brilliant-violet-510-anti-mouse-cd62l-antibody-8162

CX3CR1:
https://www.biolegend.com/de-at/products/brilliant-violet-785-anti-mouse-cx3cr1-antibody-12119

Ly6C:
https://www.biolegend.com/de-at/products/fitc-anti-mouse-ly-6c-antibody-4896
https://www.biolegend.com/de-at/products/pacific-blue-anti-mouse-ly-6c-antibody-6024

TNF:
https://www.biolegend.com/de-at/products/fitc-anti-mouse-tnf-alpha-antibody-976

Ki-67:
https://www.biolegend.com/de-at/products/brilliant-violet-605-anti-mouse-ki-67-antibody-8983

CXCR3:
https://www.biolegend.com/de-at/products/brilliant-violet-650-anti-mouse-cd183-cxcr3-antibody-9384

CD43:
https://www.biolegend.com/de-at/products/percp-cyanine5-5-anti-mouse-cd43-activation-associated-glycoform-antibody-4297

Zombie Aqua:
https://www.biolegend.com/de-at/products/zombie-aqua-fixable-viability-kit-8444

EOMES:
https://www.thermofisher.com/antibody/product/EOMES-Antibody-clone-Dan11mag-Monoclonal/25-4875-82

IL-2:
https://www.thermofisher.com/antibody/product/IL-2-Antibody-clone-JES6-5H4-Monoclonal/12-7021-82

MHC class II tetramer for the PADRE epitope (AKFVAAWTLKAA):
https://tetramer.yerkes.emory.edu/reagents/4072

MHC class I tetramers were validated inhouse.

Horse Radish Peroxidase (HRP) conjugated anti-mouse IgG secondary antibody:
https://www.southernbiotech.com/goat-anti-mouse-igg-human-ads-hrp-1030-05

Eukaryotic cell lines

Policy information about cell lines

Cell line source(s)	VeroE6 cells: ATCC CRL-1586
Authentication	The cell line was not authenticated.
Mycoplasma contamination	The cell line tested negative for Mycoplasma.
Commonly misidentified lines (See ICLAC register)	-

Animals and other organisms

Policy information about studies involving animals; ARRIVE guidelines recommended for reporting animal research

Laboratory animals	- Wild-type C57BL/6 mice were obtained from Charles River Laboratories, Jackson Laboratory or Janvier Labs.<br>- K18-hACE2 transgenic mice, expressing the human ACE2 receptor (hACE2) under control of the cytokeratin 18 (K18) promoter, were obtained from the Jackson Laboratory (B6.Cg-Tg(K18-ACE2)2Prlmn/J), and bred in-house.<br>- The OT-I Hobit reporter × ROSA26-eYFP LT mice were obtained from Sanquin Research, Amsterdam.<br><br>Animals were housed in individually ventilated cages under specific-pathogen free conditions at the animal facility at the Leiden University Medical Center (LUMC) at 20°C -22°C, a humidity of 45-65% RV and a light cycle of 6:30h-7:00h sunrise, 07:00h-18:00h daytime and 18:00h-18:30h sunset.
Wild animals	This study did not involve wild animals.
Field-collected samples	This study did not involve samples collected from the field.
Ethics oversight	All animal experiments were approved by the Animal Experiments Committee of LUMC and performed according to the recommendations and guidelines set by LUMC and by the Dutch Experiments on Animals Act.

Note that full information on the approval of the study protocol must also be provided in the manuscript.

Flow Cytometry

Plots

Confirm that:

- [x] The axis labels state the marker and fluorochrome used (e.g. CD4-FITC).
- [x] The axis scales are clearly visible. Include numbers along axes only for bottom left plot of group (a 'group' is an analysis of identical markers).
- [x] All plots are contour plots with outliers or pseudocolor plots.
- [x] A numerical value for number of cells or percentage (with statistics) is provided.

Methodology

Sample preparation	Peripheral blood was collected from the tail vein. Splenocytes were obtained by mincing the tissue through cell strainers. Blood cells and splenocytes were depleted of erythrocytes using ammonium chloride lysis buffer. To remove remaining circulating blood cells from the liver and lungs, mice were perfused with 20 ml PBS containing 2 mM EDTA. Next, liver and lungs were cut into small pieces using surgical knives, and the tissue was resuspended in 3.5 ml or 1 ml, respectively, of IMDM containing 250 U/ml collagenase type 1-A (C2674, Sigma) and 20 µg/ml DNase I (D5025, Sigma). After incubation with collagenase/DNase for 25 minutes at 37°C, liver and lung tissue was dissociated into single-cell suspensions using 70 µm cell strainers, and subsequently lymphocytes were isolated using a Percoll (GE Healthcare) gradient.
Instrument	BD Fortessa flow cytometer (BD Biosciences)<br>Aurora Cytek spectral analyzer
Software	Flowjo software (TreeStart) and OMIQ data analysis software
Cell population abundance	No sorting was performed.
Gating strategy	Lymphocyte populations were gated by FSC-A/SSC-A gates, followed by gating for single cells based on FSC-A/FSC-H gates. Live cells were then selected by exclusion of cells staining positive for live/dead fixable Zombie Aqua stain. Live cells were further gated by CD3/CD8a of CD3/CD4. Antigen-specific CD4/CD8 T cells were then selected by tetramer positivity.<br><br>Expression of phenotypic markers on antigen-specific CD8 T cells was analyzed as indicated in the figures.

- [x] Tick this box to confirm that a figure exemplifying the gating strategy is provided in the Supplementary Information.
